# Comparative Analysis of Korean Nasal Morphology Using Cone-Beam Computed Tomography

**DOI:** 10.3390/healthcare12181839

**Published:** 2024-09-13

**Authors:** Jeong-Hyun Lee, Jong-Tae Park

**Affiliations:** 1 Department of Oral Anatomy, Dankook Institute for Future Science and Emerging Convergence, Dental College, Dan-Kook University, Cheonan 31116, Republic of Korea; 911105jh@dankook.ac.kr; 2 Department of Bio Health Convergency Open Sharing System, Dan-Kook University, Cheonan 31116, Republic of Korea

**Keywords:** nasal, 3D modeling, facial index, nasal index, nose

## Abstract

Background/Objectives: Nasal morphology is a significant aspect of facial anatomy and is often used for forensic identification and aesthetic surgery. This study aims to compare nasal dimensions based on sex, facial index (FI), and nasal index (NI) using cone-beam computed tomography (CBCT) and 3D modeling. Methods: To observe differences in nasal dimensions by sex and analyze the relationships between facial shapes (FI) and nasal forms (NI), a total of 100 participants (50 males, 50 females) in their 20s were selected from Dankook University Dental Hospital. CBCT scans were performed, and 3D models were created using Mimics software (version 22.0). The measurement items included the alaria distance between (AL), the distance between N (nasion) and SN (subnasale), the distance between N (nasion) and PRN (pronasale), and the distance between SN (subnasale) and PRN (pronasale). A *T*-test was used for the sex-based analysis of the nasal dimensions, and the facial index- and nasal index-based nasal dimensions were analyzed using a one-way ANOVA with Scherffe’s post hoc test. Additionally, all the statistical analyses were performed using SPSS software (version 23.0). Results: The results indicated that males generally have larger nasal dimensions than females. Additionally, the mesoprosopic facial type (round face) showed the largest nasal dimensions in the FI classification, while the platyrrhine nasal type (broad and short nose) exhibited the largest dimensions in the NI classification. Conclusions: This study demonstrates that the nasal size varies significantly with sex, facial shape, and nasal form. The findings can contribute to forensic identification and provide valuable data for clinical practices in facial reconstruction and nasal surgery.

## 1. Introduction

In forensic research, determining the sex of unidentified skeletal remains is crucial [1]. Sex analysis is essential, particularly in criminal investigations, historical research, and disaster sites. Sex analysis primarily relies on anatomical features such as the pelvic structure, nostril size, and prominent tubercles [2,3]. However, when these areas are damaged, accuracy may decrease. Traditional methods of estimating sex using specific characteristics of the pelvis or skull can be limited, especially when these areas are compromised. Therefore, methods have been developed to identify individuals by analyzing growth patterns and degenerative changes in bones, using relatively undamaged teeth and long bones [4]. Specifically, the wear patterns of teeth and ossification of long bones can be analyzed to estimate the age, making these methods commonly used. However, the accuracy of these analyses can vary significantly depending on the completeness and condition of the teeth and skeletal remains [5]. Moreover, current trends in research focus on facial reconstruction [6,7,8,9,10], aiding in the visual identification of individuals [9]. Among these methods, 3D facial recognition technology is gaining interest for its ability to objectively and accurately analyze the entire surface of the face [11]. Among facial features, the nose holds a central position and is a noticeable structure on the face [12,13]. Additionally, the nose can be indicative of race, sex, and previous studies have suggested its impact on head and neck morphology due to biological and environmental factors [14,15,16,17,18]. Therefore, nasal measurements are an important aspect of forensic identification by analyzing the nasal structure of individuals. For instance, the shape of the nose can be differentiated by measuring parameters such as the thickness, length, and width, which, when combined with other facial features, can result in highly accurate identification. These measurements can reflect racial and sex differences, allowing for precise individual identification [19]. The nose plays a significant role in forensic identification.

Facial contours are of interest in anatomy, anthropology, plastic surgery, and art, band are essential structures in forensic science for individual identification [20]. Recently, facial index (FI) classification has been used to distinguish facial shapes. the FI categorizes faces into hypereuryprosopic, euryprosopic, mesoprosopic, leptoprosopic, and hyperleptoprosopic [21,22]. Similarly, the nasal index (NI) classifies nasal shapes based on the percentage of nasal height and width [23], categorizing noses as leptorrhine (long and narrow), mesorrhine (medium), and platyrrhine (broad) [24]. While the NI was historically evaluated using skeletal remains [25], it is now being utilized in nasal plastic surgery [14,26]. Therefore, research on facial and nasal shapes is crucial.

Most previous studies have focused on sex-based comparisons [1,2,3,4,5,6,7,8,9,10,11,12,13,14,15,16,17,18,19,20,21,22,23,24,25,26], and while there have been studies on nose size, they are limited to NI classification ratios [23,24,25,26]. However, an accurate analysis is essential due to variations in the nose size based on sex, facial shape, and nasal form. Therefore, research on the relationship between nose size, sex, facial shape, and nasal form is lacking.

The purpose of this study is to compare the nasal structures of Koreans according to sex, facial index (FI), and nasal index (NI). Through this comparison, we aim to analyze differences in nasal size by sex, as well as variations in size according to facial and nasal types, using 3D imaging technology.

Additionally, this study proposes three primary hypotheses. First, males will have larger nasal sizes than females, with statistically significant differences in nasal width, height, bridge length, and tip protrusion. Second, there will be differences in nasal size according to the facial index, with individuals having a mesoprosopic (round face) facial type exhibiting larger nasal dimensions compared to those with other facial types. Third, there will be differences in nasal size according to the nasal index, with individuals possessing a platyrrhine (broad and short) nasal type having larger nasal dimensions compared to other nasal types.

## 2. Materials and Methods

### 2.1. Research Subjects

This study included 100 participants, determined using the G-Power 3.1 (HHU, Dusseldorf, Germany) program. The following parameters were set: test family: *T*-tests; statistical test: means; difference between two independent means (two groups); effect size d 0.8; α err prob 0.05; power (1-β err prob) 0.95. A total of 84 subjects were found: 42 males and 42 females. In addition, 16 subjects were added, and 100 subjects were selected. The participants were composed of patients in their 20s (50 males and 50 females) who visited Dankook University Dental Hospital (IRB approval no. DKUDH IRB 2020-01-007). To minimize measurement errors in terms of nasal size, patients were selected with no missing teeth, facial asymmetry, nasal septal deviation, or systemic diseases. In addition, the data provided were retrospective analyses, and the Dankook University IRB requested an exemption from consent form and approved the process, so consent was not required.

### 2.2. Methods

#### 2.2.1. CBCT Data

CBCT data were captured by the same technician, ensuring the Frankfurt horizontal plane was parallel to the ground to minimize nasal dimension variations. Cone-beam computed tomography (CBCT) scans were performed using the Alphard 3030 system (Asahi, Kyoto, Japan) with parameters set at 0° gantry angle, 120 kV, auto mA, slice increment of 0.39 mm, slice thickness of 0.39 mm, slice pitch of 3, scanning time of 4 s, and a matrix of 512 px × 512 px, FOV 200 mm × 200 mm (20 × 20 cm^2^). The CBCT data were subsequently provided in DICOM format.

#### 2.2.2. Three-Dimensional Modeling

The Mimics 3D program (version 22.0, Materialise, Leuven, Belgium) was used to extract 3D patient data from the provided DICOM files. Three-dimensional modeling was conducted in three directions (coronal view, sagittal view, frontal view) to enhance accuracy. The Hounsfield Unit (HU) values were adjusted to match the skull and facial soft tissues, and 3D models were generated.

(1) Three-Dimensional Skull Modeling

For 3D skull modeling, masking was performed with a minimum of 500 HU and a maximum of 3071 HU. Unnecessary tissues and bones were removed using the Edit Mask function, and the completed data were converted to STL files using the Calculate Part function.

(2) Three-Dimensional Facial Soft Tissue Modeling

The 3D facial soft tissue modeling involved masking with a minimum of −390 HU and a maximum of 160 HU. Unnecessary bones were removed using the Edit Mask function, and the completed data were converted to STL files using the Calculate Part function.

#### 2.2.3. Measurement Items

All measurements were conducted after ensuring the Frankfurt horizontal line was horizontal. The highest point was marked using the Analyze Point function for accurate measurements. Distances between marked points were measured twice by Park and Lee, and the average values were used for reliability evaluation (Cronbach’s α = 0.623). Facial and nasal indices were calculated based on the measured data.

(1) Description of Reference Points

The following landmarks were used to assess nasal dimensions and facial indices (Table 1):

(2) Facial Index (FI)

**Figure 1:**
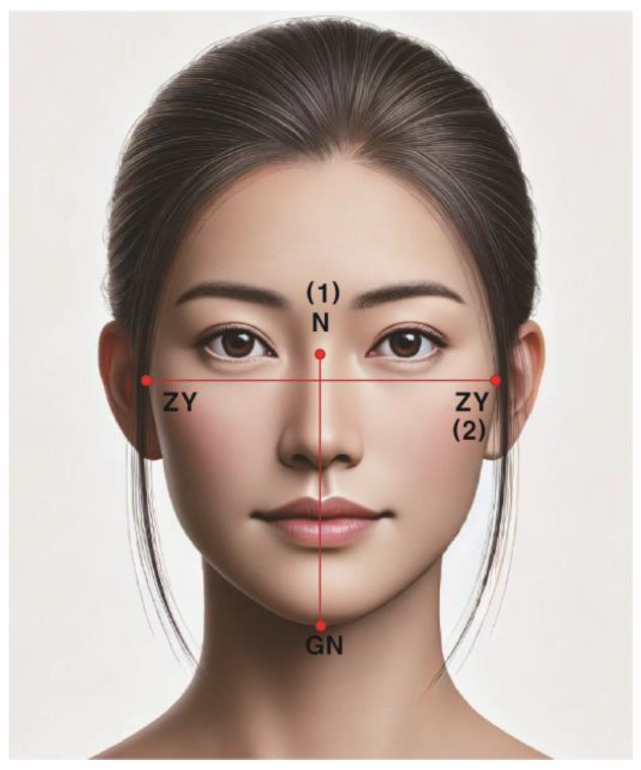
Facial index measurement items. (1) Facial height (FH): the distance between the nasion (N) and the gnathion (GN); (2) facial width (FW): the bizygomatic distance from right to left zygion (ZY).

FI was classified using the formula facial height (FH)/facial width (FW) × 100 [26]. FH was measured using the distance between the nasion (N) and gnathion (GN), and FW was measured as the bizygomatic width (zygion–zygion) (Figure 1).

(3) Nasal Index (NI)

**Figure 2:**
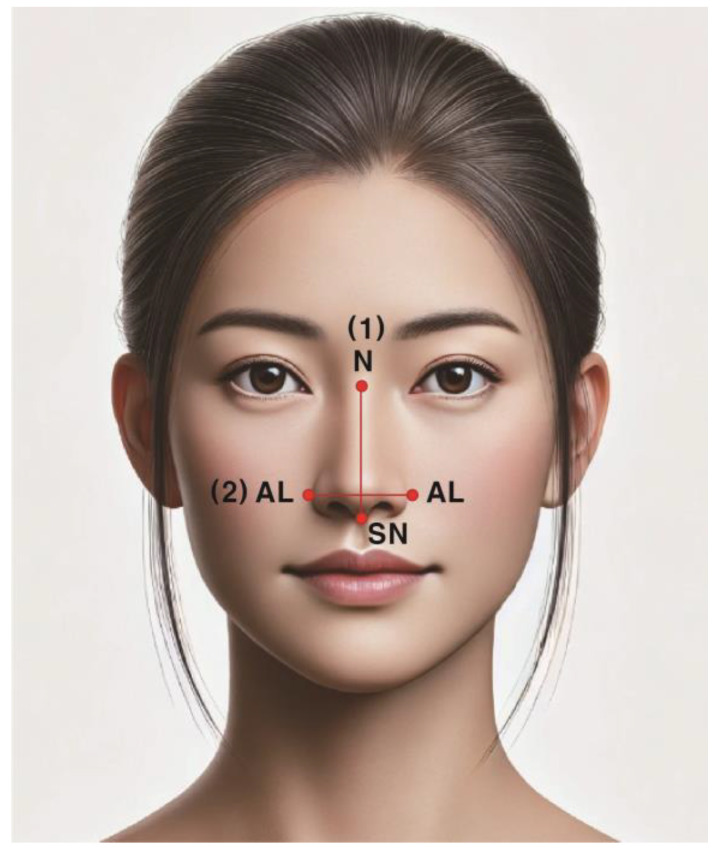
Nasal index measurement items. (1) Nasal height (NH): the distance between the nasion (N) and the subnasale (SN); (2) nasal width (NW): the distance between each alaria (AL).

NI was classified using the formula nasal width (NW)/nasal height (NH) × 100 [23]. NW was measured as alaria (AL) distance, and NH was measured as the distance between nasion (N) and subnasale (SN) (Figure 2).

(4) Specific Measurement Items

**Figure 3:**
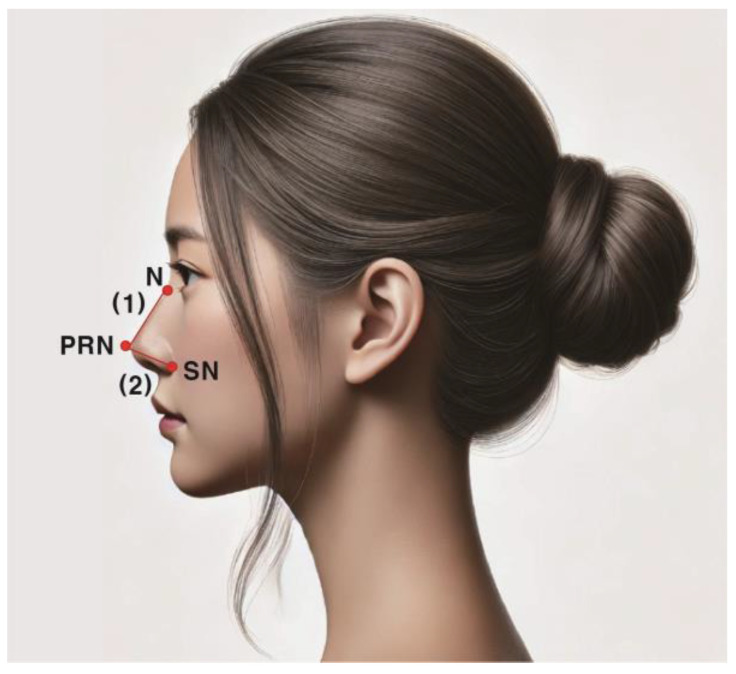
Nasal size measurements items. (1) Nasal bridge length: the distance between the nasion (N) and the pronasale (PRN); (2) nasal tip protrusion: the distance between the subnasale (SN) and the pronasale (PRN).

The measurements for nasal dimensions are detailed in Table 2 (Figure 3).

#### 2.2.4. Statistics

The measurement items were analyzed using the SPSS program (version 23.0, IBM Corporation, Armonk, NY, USA). Statistical methods included a *T*-test for sex-based analysis of nasal dimensions and a one-way ANOVA with post hoc Scheffe test for facial index- and nasal index-based analysis of nasal dimensions. All statistical analyses were conducted with a 95% confidence interval, and the significance level was set at 0.05.

## 3. Results

### 3.1. Facial Index Classification

The results showed 75 individuals were classified as hypereuryprosopic (very broad face) with a facial index below 80%, 20 individuals as euryprosopic (broad face) with a facial index below 80–85%, and 5 individuals as mesoprosopic (round face) with a facial index between 85 and 90%. No observations of leptoprosopic (long face) or hyperleptoprosopic (very long face) were made (Table 3).

### 3.2. Nasal Index Classification

The results showed 10 individuals were classified as leptorrhine (long and narrow) with a nasal index below 55–69.9%, 76 individuals as mesorrhine (moderate shape) with a nasal index below 70–84.9%, and 14 individuals as platyrrhine (broad and short) with a nasal index below 85–99.9%. No observations of hyperleptorrhine (very narrow) or hyperplatyrrhine (very broad/wide) were made (Table 4).

### 3.3. Analysis of Nasal Dimensions Based on Sex

The analysis of the differences in the nasal dimensions based on sex is presented in Table 5. The AL-AL value was 39.33 in the males and 36.70 in the females. The N-SN value was 51.91 in the males and 47.74 in the females. The N-PRN value was 40.88 in the males and 37.16 in the females. The SN-PRN values was 21.31 in the males and 19.70 in the females. Significant differences were observed in the AL-AL (*p* < 0.001), N-SN (*p* < 0.001),N-PRN (*p* < 0.001), and SN-PRN (*p* < 0.001) values, with the males generally having larger dimensions compared to the females.

### 3.4. Analysis of Nasal Dimensions Based on Facial Index (FI) Classification

The analysis of differences in the nasal dimensions based on the FI classification is presented in Table 6. The AL-AL value was hypereuryprosopic 37.92, euryprosopic 38.10, and mesoprosopic 39.17. The N-SN value was hypereuryprosopic 48.8, euryprosopic 52.87, and mesoprosopic 42.27. The N-PRN value was hypereuryprosopic 38.03, euryprosopic 41.92, and mesoprosopic 42.27. The SN-PRN value was hypereuryprosopic 20.27, euryprosopic 21.13, and mesoprosopic 21.55. Significant differences were observed in the N-SN (*p* < 0.001) and N-PRN (*p* < 0.001) values, with the mesoprosopic type having the largest dimensions.

### 3.5. Analysis of Nasal Dimensions Based on Nasal Index

The analysis of differences in the nasal dimensions based on the nasal index is presented in Table 7. The AL-AL value was 37.92 for leptorrhine, 38.02 for mesorrhine, and 39.98 for platyrrhine. The N-SN value was 53.94 for leptorrhine, 50.08 for mesorrhine, and 45.50 for platyrrhine. The N-PRN value was 43.21 for leptorrhine, 39.26 for mesorrhine, and 34.72 for platyrrhine. The SN-PRN value was 20.56 for leptorrhine, 20.55 for mesorrhine, and 20.24 for platyrrhine. Significant differences were observed in the AL-AL (*p* < 0.001), N-SN (*p* < 0.001), and N-PRN (*p* < 0.001) values, with the platyrrhine type having the largest dimensions.

## 4. Discussion

Facial reconstruction is a method used in forensic medicine to identify skulls [6]. Among the facial features, the nose is crucial in facial reconstruction as the area around the center of the nose remains fixed for eye recognition [7]. Although research related to the nose is ongoing, most studies focus on sex comparisons and the proportional aspects of nasal shape [1,2,3,4,5,6,7,8,9,10,11,12,13,14,15,16,17,18,19,20,21,22,23,24,25,26,27,,28,29,30,31]. However, variations in facial structure, size, shape, and proportions exist [27,28]. Therefore, research on nasal size comparisons based on sex, facial shape, and nasal form is essential. Thus, in this study, we aimed to investigate differences based on sex, facial shape, and nasal form using a 3D program.

To achieve this, three primary hypotheses were proposed. First, it was hypothesized that males would have larger nasal sizes than females, with statistically significant differences expected in the nasal width, height, bridge length, and tip protrusion. Second, it was hypothesized that there would be differences in the nasal size according to the facial index, with individuals having a mesoprosopic (round face) type expected to exhibit larger nasal dimensions than those with other facial types. Third, it was hypothesized that the nasal size would also differ according to the nasal index, with individuals possessing a platyrrhine (broad and short) nasal type anticipated to have larger nasal dimensions than those with other nasal types.

The results of this study partially or fully supported these hypotheses. For the first hypothesis, it was proposed that the males would have larger nasal sizes than the females, with statistically significant differences in the nasal width, height, bridge length, and tip protrusion. In the comparison of the nasal size based on sex, significant differences were observed in the AL-AL (*p* < 0.001), N-SN (*p* < 0.001), N-PRN (*p* < 0.001), and SN-PRN (*p* < 0.001) values, indicating that the males had larger noses than the females. This was consistent with the first hypothesis. This aligns with previous studies [28,29,30,31]. According to the study by Zhuang et al. [28], a comparison of the nasal sizes between African Americans and Caucasians revealed that males generally have larger noses than females, consistent with the findings of the present study. The study also reported that African Americans tend to have shorter and wider noses compared to Caucasians. Similarly, Zaidi et al. [29] found that males have larger nasal sizes than females. Additionally, when evaluating the nasal size across West Africans, East Asians, South Asians, and Northern Europeans, the order of nasal size from largest to smallest was that of West Africans, Northern Europeans, South Asians, and East Asians. According to LoMauro et al. [32], males exhibit larger lungs and longer thoracic walls than females. Additionally, studies [33] indicate that males have larger bodies and nasal sizes with a narrower nasal floor. Therefore, it appears that males generally have larger dimensions in terms of nasal width and height.

The results of the facial index (FI) classification revealed three types: hypereuryprosopic, euryprosopic, and mesoprosopic. For the second hypothesis, it was proposed that there would be differences in nasal size according to the facial index, with individuals having a mesoprosopic (round face) facial type exhibiting larger nasal dimensions compared to those with other facial types. As a result, the mesoprosopic (round face) type showed the largest nose in terms of the AL-AL (*p* > 0.05), N-SN (*p* < 0.05), N-PRN (*p* < 0.05), and SN-PRN (*p* > 0.05) values. This was consistent with the first hypothesis. A study by Ryu et al. [34] found a significant correlation between facial tissues, nasal openings, and the overall facial and skull shape. Additionally, Gupta et al.’s study [35] suggests that the mesoprosopic type has the largest nasal airway volume. Therefore, the mesoprosopic types with FI indices of 85% and 90% exhibit larger dimensions in terms of the nasal width and height compared to the other types.

The analysis based on the nasal index revealed three types: leptorrhine, mesorrhine, and platyrrhine. For the third hypothesis, it was proposed that there would be differences in the nasal size according to the nasal index, with the individuals possessing a platyrrhine (broad and short) nasal type having larger nasal dimensions compared to those with other nasal types. As a result, the platyrrhine type showed the largest nose in terms of the AL-AL (*p* < 0.001), N-SN (*p* < 0.001), N-PRN (*p* < 0.001), and SN-PRN (*p* > 0.05) values. This was consistent with the first hypothesis. According to Leong et al.’s study [36], the nasal shape and size are influenced by climate, with the platyrrhine type being associated with hot and humid climates, leading to a flattened form for efficient air regulation [37]. Therefore, platyrrhine types exhibit larger nasal dimensions due to their flatter nasal form.

This study yielded significant results from analyzing the size and shape of the nose, but it has several limitations. First, the study was conducted exclusively on a single ethnic group (Korean) of patients in their 20s, which restricts the generalizability of the findings to other age groups and ethnic populations. Additionally, the study did not provide information on the participants’ body size or height, factors that could influence the nasal size due to their association with respiratory requirements. The lack of consideration for these variables may limit the scope of the findings. Second, the study focused on nasal size using specific facial (FI) and nasal (NI) indices, without fully incorporating other facial features or the overall cranial morphology.

Despite these limitations, this study offers valuable baseline data on variations in the nasal size by gender, facial shape, and nasal form, serving as a foundation for further research in this area. Future studies should include more diverse population samples and account for potential confounding factors such as ethnicity, body size, and comprehensive cranial morphology to enhance the generalizability and applicability of the findings.

## 5. Conclusions

In this study, a comparative analysis of nasal dimensions based on sex, the FI, and nasal index was conducted. The results indicated that males generally have larger noses than females. The mesoprosopic types with round faces had the largest nose in terms of the FI, while the platyrrhine types with broad and short nasal forms had the largest nose based on the nasal index. These findings suggest that the nasal size varies not only based on sex but also on the facial shape and nasal form. Future research in this area is anticipated to include not only sex- but also facial and nasal shape-related studies. Moreover, the results could potentially contribute to forensic facial reconstruction and aesthetically informed nasal surgeries in clinical treatments.

## Figures and Tables

**Table 1:** Nasal dimension measurement *landmark*.

Landmark	Definition
Zygion (ZY)	The most lateral point on the zygomatic arch (cheekbone) on both sides of the face. It represents the widest point of the face and is used to measure facial width (bizygomatic width).
Nasion (N)	The midpoint of the nasofrontal suture, located at the intersection where the frontal bone meets the nasal bones. It serves as a key reference point for measuring nasal height and facial height.
Gnathion (GN)	The lowest point on the midline of the mandible (chin), marking the lowest edge of the face. It is used in conjunction with the nasion to measure total facial height.
Subnasale (SN)	The point at the base of the nasal septum where the nasal columella meets the upper lip. It is used to measure nasal height and nasal tip protrusion.
Pronasale (PRN)	The most anterior point on the tip of the nose. It serves as a reference for measuring nasal tip protrusion from the subnasale.
Alare (AL)	The most lateral point on each alar contour (nostril wings) of the nose. This landmark is used to measure nasal width (distance between the alare points on both sides).

**Table 2:** Nasal dimension measurement items.

Parameter	Definition
Nasal width (AL-AL)	Alaria (AL) distance
Nasal height (N-SN)	Nasion (N) to subnasale (SN) distance
Nasal bridge length (N-PRN)	Nasion (N) to pronasale (PRN) distance
Nasal tip protrusion (SN-PRN)	Subnasale (SN) to pronasale (PRN) distance

**Table 3:** Facial index classification.

Facial Index	Range of FI	N
Hypereuryprosopic (very broad face)	80%	75
Euryprosopic (broad face)	80~85%	20
Mesoprosopic (round face)	85~90%	5

**Table 4:** Nasal index classification.

Nasal Index	Range of FI	N
Leptorrhine (long and narrow)	55~69.9%	10
Mesorrhine (moderate shape)	70~84.9%	76
Platyrrhine (broad and short)	85~99.9%	14

**Table 5:** Analysis of nasal dimensions based on sex.

Dimension	Sex	N	Mean (SD)	F	t	*p*
AL-AL (mm)	male	50	39.33 (2.43)	0.273	5.398	0.000 *
	female	50	36.70 (2.45)			
N-SN (mm)	male	50	51.91 (3.15)	0.506	6.406	0.000 *
	female	50	47.74 (3.36)			
N-PRN (mm)	male	50	40.88 (3.68)	0.418	5.294	0.000 *
	female	50	37.16 (3.35)			
SN-PRN (mm)	male	50	21.31 (2.26)	0.737	3.778	0.000 *
	female	50	19.70 (1.99)			

Data are mean (standard deviation values); *p*-values were obtained by *T*-test (* *p* < 0.001).

**Table 6:** Analysis of nasal dimensions based on FI classification.

Dimension	FI Classification	N	Mean (SD)	F	*p*
AL-AL (mm)	hypereuryprosopic	75	37.92 (2.86)	0.483	0.619
	euryprosopic	20	38.10 (2.49)		
	mesoprosopic	5	39.17 (2.52)		
N-SN (mm)	hypereuryprosopic	75	48.8 (3.77)	13.212	0.000 *
	euryprosopic	20	52.87 (2.25)		
	mesoprosopic	5	53.03 (1.74)		
N-PRN (mm)	hypereuryprosopic	75	38.03 (3.70)	11.291	0.000 *
	euryprosopic	20	41.92 (3.52)		
	mesoprosopic	5	42.27 (1.82)		
SN-PRN (mm)	hypereuryprosopic	75	20.27 (2.27)	1.736	0.182
	euryprosopic	20	21.13 (2.20)		
	mesoprosopic	5	21.55 (1.94)		

Data are mean (standard-deviation values); *p*-values were obtained by one-way ANOVA (* *p* < 0.001).

**Table 7:** Analysis of nasal dimensions based on nasal index.

Dimension	Nasal Index	N	Mean (SD)	F	*p*
AL-AL (mm)	leptorrhine	10	35.24 (2.03)	10.169	0.000 *
	mesorrhine	76	38.02 (2.64)		
	platyrrhine	14	39.98 (2.23)		
N-SN (mm)	leptorrhine	10	53.94 (2.19)	20.288	0.000 *
	mesorrhine	76	50.08 (3.43)		
	platyrrhine	14	45.50 (2.96)		
N-PRN (mm)	leptorrhine	10	43.21 (2.28)	18.991	0.000 *
	mesorrhine	76	39.26 (3.63)		
	platyrrhine	14	34.72 (2.58)		
SN-PRN (mm)	leptorrhine	10	20.56 (2.20)	0.111	0.895
	mesorrhine	76	20.55 (2.40)		
	platyrrhine	14	20.24 (1.52)		

Data are mean (standard-deviation values); *p*-values were obtained by one-way ANOVA (* *p* < 0.001).

## Data Availability

Original data are available upon request to the corresponding author.
